# Editorial: Microbial mechanisms for the behavior of toxic metals in soil

**DOI:** 10.3389/fmicb.2023.1269230

**Published:** 2023-09-18

**Authors:** Xinyue Wu, Chaolei Yuan, Weimin Sun, Zhimin Xu

**Affiliations:** ^1^College of Resources and Environment, Zhongkai University of Agriculture and Engineering, Guangzhou, China; ^2^School of Agriculture, Sun Yet-sen University, Shenzhen, China; ^3^Institute of Eco-Environmental and Soil Sciences, Guangdong Academy of Sciences, Guangzhou, China

**Keywords:** soil, microbial processes, toxic metals, metal resistance genes, risk index

The rapid development of industry has led to increasingly severe soil pollution by toxic metals, attracting growing public concern. Microbes play a vital role in the biogeochemical cycling of toxic metals, because they can drive redox reactions that alter the speciation of metals, influencing their mobility and toxicity (Zhang et al., 2022). Furthermore, microbial cells can adsorb metals due to the presence of various functional groups on the cell wall (Priya et al., 2022). Understanding the mechanisms of transport and transformation of toxic metals as affected by microbial activities are crucial for the remediation and management of polluted soils.

Anthropogenic sources of toxic metals include wastewater, solid waste (e.g., sludge), dust, phosphorus fertilizers, etc. The sludge from wastewater treatment plant often contains high concentrations of toxic metals. If applied without pre-treatment, sludge may cause soil pollution. To address this Research Topic, aerobic composting techniques can be used for the treatment of sludge. Firstly, the relationship of microbial community succession with the composting efficiency of sludge and the detoxification of toxic metals was investigated by Han et al.. They found that utilizing a microbial inoculum yielded the most effective composting and detoxifying performance, compared to other treatments. In addition, throughout various composting phases, distinct groups of dominant bacteria thrived and perhaps contribute to the detoxification of toxic metals. Besides, toxic metals, microbial community composition, and metal resistance genes (MRGs) in river sediments were analyzed by Fu et al.. They found no very clear relationships between microbial diversity and toxic metal concentration. Some bacteria were found abundant, likely due to their possession of MRGs that can help combat elevated metal concentrations. Man-made pollution was also noted to influence MRG distribution, adding valuable insights for the impact of toxic metals on the microbial ecology in river sediments (Ma et al., 2021).

The potential ecological risk index (RI) is widely used tool for soil pollution assessment (Wei et al., 2022). Here, the response of soil microbial community structure and function to different RI levels of Pb and Cd were investigated by Li, Chen et al.. They observed that bacterial abundance and diversity were significantly lower under elevated RI levels, while fungal abundance and diversity showed an opposite trend. These findings may help future risk assessments of Pb and Cd pollution using microbial parameters. Soil contamination often involves multiple pollutants, as seen in contaminated soils around smelting factories. Shen et al. investigated bacterial and fungal communities in soil samples collected inside and outside a coking plant in China. They identified the biomarkers of soil microbes under prolonged exposure of multiple toxic metals. Especially, they detected elevated soil Hg levels and identified 25 sensitive biomarkers. The authors also explored the co-occurrence pattern of soil bacteria and fungi. The results shed light to the response of soil microbiome to the stress of multiple toxic metals in typical contaminated soils. Li, Meng et al. examined the metabolic capacity of microbial populations in metal-rich soils using metagenomic analysis. Microorganisms resistant to toxic metals, with potential for bioremediation, were identified. Moreover, the genes related to metal resistance and transformation in microorganisms, especially key species, were examined. This study may help understand the cytotoxicity of toxic metals. Finally, Liu et al. found that toxic metal pollution led to the decrease in soil nutrients and bacterial diversity, especially in heavily polluted zones. The relative abundances of some major bacterial groups changed significantly due to pollution. Soil properties primarily explain these changes. Furthermore, the shift in bacterial community affected soil's ecological functions.

Despite notable progresses, the microbial mechanisms underlying toxic metal behavior in soil are yet to be further explored. Firstly, how microorganisms adapt to environments with elevated toxic metal concentrations, through mechanisms such as tolerance and detoxification, requires further investigation. Then, microbe-based methods can be developed for the remediation of toxic-metal contaminated soils. Moreover, a more accurate modeling is needed to describe and predict the dynamic interactions between microbial activity and the behavior of toxic metals in soil. Finally, the development of reliable methods is desired for the determination of key microbes that affect the chemical processes of toxic metals in soil.

## Author contributions

XW: Writing—original draft. CY: Formal analysis, Writing—review and editing. WS: Visualization, Writing—review and editing. ZX: Conceptualization, Project administration, Writing—review and editing.
